# Do weather changes influence physical activity level among older adults? – The Generation 100 study

**DOI:** 10.1371/journal.pone.0199463

**Published:** 2018-07-06

**Authors:** Nils Petter Aspvik, Hallgeir Viken, Jan Erik Ingebrigtsen, Nina Zisko, Ingar Mehus, Ulrik Wisløff, Dorthe Stensvold

**Affiliations:** 1 Department of Sociology and Political Science, Faculty of Social and Educational Sciences, Norwegian University of Science and Technology (NTNU), Trondheim, Norway; 2 K.G. Jebsen Center of Exercise in Medicine at Department of Circulation and Medical Imaging, Faculty of Medicine, Norwegian University of Science and Technology (NTNU), Trondheim, Norway; 3 School of Human Movement & Nutrition Sciences, University of Queensland, Brisbane, Australia; Columbia University, UNITED STATES

## Abstract

**Introduction:**

Understanding how individual and environmental factors impact physical activity (PA) level is important when building strategies to improve PA of older adults. No studies have examined how hour-to-hour weather changes influence PA in older adults or how the association between weather and PA eventually is related to cardiorespiratory fitness (CRF) measured as peak oxygen uptake. The aim of this study was therefore to examine how hour-to-hour changes in weather effects hour-to-hour PA in a cohort of Norwegian older adults across CRF levels, gender and seasons.

**Methods:**

PA was assessed objectively in 1219 older adults (70–77 years, 51% females) using the Actigraph GT3X+ accelerometer, and quantified as counts·min^-1^ (CPM). Weather (Norwegian meteorological Institute) and CRF (MetaMax II) were measured objectively. Panel data analysis added a longitudinal dimension when 110.888 hours of weather- and PA data were analyzed.

**Results:**

Older adults had a higher PA level in warmer (597 CPM) than colder months (556 CPM) (p<0.01). Fixed effects regression-models revealed that increasing temperatures (per hour) influenced PA positively in both colder and warmer months (all, p<0.01), with greater influence in fitter vs. less fit participants (p<0.01). In warmer months, increasing precipitation negatively influenced PA in both unfit females and unfit males (p<0.01). In colder months, increasing precipitation positively influenced PA for moderately fit to fit males (p<0.01), but not for females and unfit males.

**Conclusion:**

To the best of our knowledge, this is the first study to examine the association between weather conditions and objectively-measured-PA among Norwegian older adults. Our findings demonstrates that unfit older adults will be less likely to participate in PA when the weather is unpleasant, compared to those highly fit. The data suggests that the impact of weather should not be ignored when planning public health strategies for increasing PA among older adults.

## Introduction

Adopting physical activity (PA), even in older age, is beneficial for health [1, 2]. Yet few older adults (+65 year of age) meet the current PA recommendation of having 150 minutes of moderate-to-vigorous PA (MVPA) per week [3–6]. To increase PA participation in this population, it is important to understand the broad range of individual and environmental factors that could impact PA levels [7–10]. Fitness, gender, social support, living situation and environment have all been found to influence PA in older adults [7, 10]. However, in studies examining objectively measured PA, 70–80% of the variance in PA remains unexplained [7, 11]. This clearly suggests that PA is a complex phenomenon, and weather conditions might be one influential factor [12]. The fact that outdoor recreational walking is the most commonly reported mode of PA among older adults, clearly underpins the relevance of weather when examining PA in this age group [12–14].

Most studies that have examined the relation between weather and PA among older adults have analyzed seasonal differences, demonstrating higher PA levels in warmer compared to colder months [7, 12, 15, 16]. In contrast, no association between PA levels and seasonality was found in a study performed in Perth (Australia), with a stable temperate climate [17]. These findings suggested that PA research should acknowledge the specific geographical location when examining the association between weather and PA.

To eliminate possible seasonal confounders recent studies have focused on day-to-day or hour-to-hour variations when examining the relation between weather and PA [18]. In a UK study on 4162 older adults, poor weather conditions, such as heavy rain and low temperatures, were associated with lower PA levels [19]. In a Dutch study on 3248 older adults, the participants walked more at higher temperatures and absence of rain [18]. Similar results have been found among Scottish and German older adults [20, 21]. However, no studies have examined the effect of weather on PA participation among older adults in the Nordic countries, such as Norway, with severe winters, no dry season, with cool, short summers and strong seasonality (subarctic climate).

When examining how weather influences PA in older adults, it is important to acknowledge that weather might influence older individuals differently. For example, in a study of Canadian adults, those with stronger commitment to PA were more willing to accept some unpleasant weather [16]. Similarly, Salmon et al. [22] reported that individuals who found exercise most enjoyable were also least likely to cite weather as a barrier for PA.

The aim of the current study was to investigate how hour-to-hour changes in weather (precipitation and temperature) affect PA among older Norwegian adults across seasons and gender. Additionally, by including older adult`s level of cardiorespiratory fitness (CRF), we aimed to examine if weather influences PA participation differently in fit compared to moderately fit and unfit individuals.

## Methods

### Study design and population

This study was part of the larger randomized controlled trial entitled the Generation 100 study (ntnu.edu/cerg/generation100) [23, 24]. All males and females born between years 1936 and 1942, with a permanent address in the municipality of Trondheim, Norway were invited to participate. Detailed description of the Generation 100 study protocol has been published elsewhere [24]. Individuals with valid measures of PA and CRF (77.4% of the larger sample of 1567) were included in the present analysis; 1219 participants (110.888 hours of PA- and weather data), 624 (51%) females, with an age range from 70 to 77 years. There were no significant differences between the analyzed sample and the Generation 100 sample (i.e. level of education and self-reported- health and PA). Descriptive characteristics of the current study participants are presented in Table 1. The present study was approved by the Regional Committees for Medical and Health Research Ethics (REK 2016/1441 B), and addresses baseline data from the Generation 100 study (August 2012 to June 2013). All participants gave their written informed consent. The study was conducted in conformity with the declaration of Helsinki.

**Table 1:** Participant characteristics.

n = 1219 participants	Male (n = 595)	Female (n = 624)	Gender differences
Age, yr	72.3 (±2.1)	72.5 (±2.1)	P = 0.09
Living alone (%)	12.0%	36.9%	**P<0.01**
Education (high %)	55.4%	43.7%	**P>0.01**
Good health (%)	89.7%	86.0%	P = 0.06
BMI	26.3 (±3.3)	25.5 (±3.7)	**P<0.01**
CRF	31.9 ((±6.7)	26.4 (±5.1)	**P<0.01**

Values are presented as means ± SD or percentage distributions. BMI: Body mass index (kg/m^2^). CRF; Cardiorespiratory fitness measured as VO_2peak_ (mL·kg^-1^·min^-1^).

### Measures

#### Assessment of participant characteristics

Age and sex were obtained from the National Population Registry. Age was calculated from month/year of birth and month/year of inclusion at baseline. Living situation, level of education and health status was assessed by previously described questionnaires [24].

Living alone and self-report of good health were dichotomized (no vs. yes). Level of education was dichotomized into low education (not attended college or university) vs. high education (attended college or university).

#### Assessment of physical activity

To measure overall PA in this study sample of older adults, we used Actigraph GT3X+ activity monitor (Actigraph, Pensacola, Florida, USA). Accelerometers are small sized, suitable for use across all age groups, and extensively validated [25, 26]. Actigraph GT3X+ uses a solid state triaxial accelerometer to collect motion data on three axes; vertical (Y), horizontal right–left (X) and horizontal front–back axis (Z). Acceleration from the three axes is converted into activity counts that increase linearly with the magnitude of the acceleration [27]. The epoch length was set to a 10-s interval and the outcome variable was triaxial counts·min^-1^ (CPM). The activity counts reflect the intensity of bodily movement, with higher number of counts indicating higher activity levels [28]. While intensity thresholds are commonly used to analyze PA data, overall PA per hour (CPM) was preferred in this panel study. The CPM data are not influenced by any external criteria (i.e. intensity threshold) other than wear time [29], and are, therefore, less vulnerable for methodological issues compared to applying intensity thresholds [30].

The activity monitor was placed on the participants (around the waist) the day they came in for clinical testing, and the participants were told to wear it for 7 consecutive days (including day and night). The Actilife software version 6.13.3 (Actigraph, Pensacola, Florida, USA) was used to analyze accelerometer data. All complete hours (60 minutes) of data between 6:00 a.m. and midnight were included in the analysis. Non-wear time, defined as intervals of at least 60 consecutive minutes with zero counts with allowance of 1–2 minutes with counts greater than zero, was excluded from the analysis [29]. Data were considered valid if the subject had at least 4 days of at least 10 hours·d^-1^ recorded.

#### Assessment of weather

From the Norwegian Meteorological Institute (MET Norway) we obtained hour-to-hour weather data for the same location (municipality of Trondheim) and the period of time as the PA data. MET Norway is the meteorological service for both the Military and the Civil Services in Norway, as well as the public (met.no/). Three variables from MET Norway were included in the present study; precipitation, temperature and wind velocity. All three variables were continuous with a mean hour-to-hour outcome. Precipitation was measured as mean hourly millimeter (mm), temperature was measured as mean hourly temperature (°C), and wind velocity was measured as mean hourly meter per second (m·s^−1^) (Table 1). Wind was included and treated as a control variable in the present study.

#### Assessment of season

Season was dichotomized into “colder” (November-March) and “warmer” (April-October) months. In Trondheim, colder months have higher probability of snow, ice and relatively few hours of daylight.

#### Assessment of cardiorespiratory fitness and body mass index

The MetaMax II (Cortex, Leipzig, Germany) was used to test peak oxygen uptake (VO_2peak_; mL·kg^-1^·min^-1^). The MetaMax II apparatus was tested against Douglas-bag and iron lung (Cortex, Leipzig, Germany) [31]. Testing of VO_2peak_ was initiated using inclination and speed derived from warm-up and performed as an individualized ramp protocol. The load was increased gradually (by either 2% inclination or 1 km·h^-1^) approximately every minute until exhaustion (VO_2peak_), or until the true maximal oxygen uptake (VO_2max_) was reached. VO_2max_ were considered met when participants' oxygen uptake did not increase by more than 2 mL·kg^-1^·min^-1^ in the last 30 seconds of the test (leveling off of oxygen uptake) despite increased workload and respiratory-exchange-ratio (RER) >1.05. A person’s VO_2max/peak_ was measured as the mean of the three successively highest 10-s VO_2_ registrations. Since 505 participants (41%) did not reach the VO_2max_, the term VO_2peak_ was used in the current study. Participants with cardiovascular diseases were tested under ECG monitoring, and the American College of Cardiology/American Heart Association guidelines for exercise testing of patients with known cardiovascular disease were followed [24].

Based upon a previous study in this population [13], the participants in the current study was categorized into unfit (25% lowest; Males <27.0 mL·kg^-1^·min^-1^, females <23.6 mL·kg^-1^·min^-1^), moderately fit (50% medium; males 27.0–35.6 mL·kg^-1^·min^-1^, females 23.6–29.8 mL·kg^-1^·min^-1^), and highly fit (25% highest; males high >35.6 mL·kg^-1^·min^-1^, females high >29.8 mL·kg^-1^·min^-1^) individuals.

Body mass index (BMI) was calculated as body weight (kg) divided by the squared value of height (m) (kg/m^2^). Height was measured with a mechanical telescopic measuring stadiometer (Seca 222, Hamburg, Germany). Weight was measured using bioelectrical impedance (Inbody 720, BIOSPACE, Seoul, Korea).

### Statistical procedures

All statistical analyses were performed with Stata software, version 12.1 (StataCorp LP, College Station, TX, USA). Panel data analysis was used to investigate the association between weather and PA. The panel data analysis adds a longitudinal dimension, where each unit is measured at more than one point in time [32, 33]. In this case, mean unit per hour from the first hour that the participant wore the accelerometer, to the last hour.

The Hausman test [34] was performed and it was determined that a fixed effects model, which is a more consistent model than random effects [35], best suited the data. Moreover, in most cases using panel data, the error term would be correlated over time [32, 33]. The Wooldridge test, which tests serial correlation in linear panel-data, was therefore performed [36] and identified a problem of autocorrelation in the model (P<0.05). This finding suggested the use of the Huber/White robust standard errors, which will relax the assumption that the errors are distributed identically. In addition, the cluster option was performed in the model, to relax the assumption that the error terms are independent of each other [37]. Interaction effects included in a sub analysis (precipitation, temperature, wind), did not improve the model and were therefore not included in the current study.

Regression coefficients reported in our fixed effects regression models represent the mean change in PA (CPM) for one unit of change in one of the predictor variables (precipitation: mm, temperature: °C, wind: m·s^−1^) while holding the other predictors in the model constant. *R*^*2*^ *within* reports how much precipitation, temperature and wind explains of the variance in PA. *Rho* reports how much of the variance is due to difference across time (interclass correlation) [33]. A P-value < 0.05 was required to declare statistical significance.

## Results

Out of the 1219 older adults included, 58% wore the accelerometer for 7 valid days or more, 32% wore it for 6 valid days, and 10% wore it for 4–5 days. The mean daily wear time was 964 minutes (≈16 hours); 961 minutes for females and 968 minutes for males, respectively.

A total of 110 888 hours of PA- and weather data were included in the present study. Females had a higher overall PA than males (562 vs. 588 CPM, p<0.01). Moreover, older adults were more physically active in warmer months compared to colder months (597 vs. 556 CPM, p<0.01), and highly fit older adults (672 CPM) were more active than those moderately fit (582 CPM) and unfit (468 CPM) older adults (p<0.05). Participant characteristics and descriptive statistics of weather and physical activity data are presented in Table 1 and Table 2, respectively.

**Table 2:** Descriptive statistics of weather and physical activity across season.

n = 1219, 110888 hours	Colder month (n = 631, 53833 hours)	Warmer month (n = 588, 57055 hours	Seasonal differences
**Weather conditions** ^a^			
Precipitation	0.06 (±0.2)	0.08 (±0.3)	**P<0.01**
Temperature	-2.4 (±5.8)	6.8 (±4.5)	**P<0.01**
Wind	2.9 (±1.6)	2.8 (±1.7)	**P<0.01**
**Overall PA** ^b^	555.6 (±589.9)	597.3 (±650.9)	**P<0.01**
Male	540.3 (±628.2)	589.2 (±694.7)	**P<0.01**
Female	572.4 (±544.0)	604.1 (±611.6)	**P<0.01**
**CRF categories** ^c^			
Unfit individuals	443.3 (±434.3)	488.6 (±495.2)	**P<0.01**
Moderately fit individuals	553.7 (±570.1)	613.5 (±643.7)	**P<0.01**
Highly fit individuals	651.1 (±709.5)	696.0 (±796.3)	**P<0.01**

^a^ Weather conditions: Precipitation; measured as mm, Temperature; measured as °C, Wind; measured as m·s^−1^.

^b^ Overall PA: triaxial counts·min^-1^ (mean per hour).

^c^ CRF categories: CRF measured as VO_2peak_ (mL·kg^-1^·min^-1^) and categorized into unfit (25% lowest), moderately fit (50% medium) and highly fit (25% highest) individuals.

CRF: Cardiorespiratory fitness. PA: Physical activity.

Weather explained approximately 1.2% of the variance within older adult`s PA. Approximately 10% of the variance in PA was due to changes in weather across time (interclass correlation) (Table 3). When analyzing weather and PA without controlling for season, gender and level of CRF, we observed that precipitation did not influence PA, while for every additional °C PA increased by an average of 20 CPM (p<0.01).

**Table 3:** Regression coefficients of weather and physical activity.

Total n = 1219, 110888 hours		Weather and PA across season		Season difference
	PA and weather	Colder months ^d^ (n = 631, 57055 hours)	Warmer months ^d^ (n = 588, 53833 hours)	Colder months vs. Warmer months
PA	524.7 (4.1)	597.4 (5.2)	329.2 (14.0)	
Precipitation ^*a*^	-10.2 (9.2)	38.2 (12.7)**	-20.6 (12.6)	P < 0.01
Temperature ^*a*^	20.2 (0.9)**	10.6 (0.9)**	33.3 (2.1)**	P < 0.01
Wind ^*a*^	3.7 (1.5)**	-8.4 (5.2)**	15.0 (2.2)**	P < 0.01
R^2^ within ^b^	1.2%	0.4%	2.8%	
Rho ^c^	10.3%	8.3%	10.4%	
F	3.1**	3.6**	3.6**	

^a^ Precipitation; measured as mm, Temperature; measured as °C, Wind; measured as m·s^−1^.

^b^ R^2^ within; How much precipitation, temperature and wind explains of the variance in Overall PA

^c^ Rho; How much of the variance is due to difference across time (interclass correlation).

^d^ Colder months: November-March, Warmer months: April-October

**Statistical significant (P < 0.01)

*Statistical significant (P < 0.05), ns Non-significant.

PA: Physical activity measured as triaxial counts·min^-1^ (mean per hour).

### Weather and physical activity across seasons and gender

Weather variations explained substantially more of the variance in PA in warmer months (2.8%) compared to colder months (0.4%) (Table 3). When not controlling for gender and level of CRF, PA increased with 38 CPM for every additional mm precipitation in colder months (p<0.05), while precipitation did not influence PA in warmer months. Moreover, PA increased with 11 and 33 CPM for every additional °C in colder and warmer months, respectively (all, p<0.01). Precipitation and temperature influenced PA differently between colder and warmer months (all, p<0.01).

When not controlling for season and level of CRF, PA increased with 20 CPM for every additional °C for both males and females (all, p<0.01) (Table 4). Furthermore, as precipitation increased, CPM of females decreased (-30 CPM, p<0.01).

**Table 4:** Regression coefficients of weather and physical activity across gender.

Total n = 1219, 110888 hours	Weather and PA across gender		Gender difference
	Females (n = 624, 56386 hours)	Males (n = 595, 54502 hours)	Females vs. Males
PA	527.8 (5.5)	521.7 (6.0)	
Precipitation ^a^	-30.2 (10.1)**	11.3 (15.5)	P < 0.05
Temperature ^a^	20.0 (1.3)**	20.4 (1.3)**	ns
Wind ^a^	5.8 (1.9)**	1.5 (2.3)	ns
R^2^ within ^b^	1.4%	1.0%	
Rho ^c^	11.3%	9.5%	
F	3.6**	3.6**	

^a^ Precipitation; measured as mm, Temperature; measured as °C, Wind; measured as m·s^−1^.

^b^ R^2^ within; How much precipitation, temperature and wind explains of the variance in Overall PA

^c^ Rho; How much of the variance is due to difference across time (interclass correlation).

**Statistical significant (P < 0.01)

*Statistical significant (P < 0.05), ns Non-significant.

PA: Physical activity measured as triaxial counts·min^-1^ (mean per hour).

### Weather and physical activity across cardiorespiratory fitness

For every additional mm precipitation, PA decreased with 47 CPM among unfit individuals (p<0.01) (Table 5). Precipitation did not influence PA among moderately- and highly fit individuals. PA increased with 17-, 18- and 29 CPM for every additional °C in unfit, moderately fit and highly fit individuals, respectively (all, p<0.01). The influence of temperature was significantly different between the unfit and highly fit individuals (p<0.01).

**Table 5:** Regression coefficients of weather and physical activity across categories of cardiorespiratory fitness.

Total n = 1219, 110888 hours	Weather and PA, across CRF categories ^a^			CRF difference	
	Unfit (n = 346, 27838 hours)	Moderately fit (n = 579, 55143 hours)	Highly fit (n = 294, 27907 hours)	Unfit vs. Moderately fit	Unfit vs. highly fit
PA	412.1 (6.8)	539.0 (5.8)	607.3 (9.1)		
Precipitation ^b^	-47.3 (11.3)**	-13.7 (11.4)	-25.0 (23.0)	P < 0.05	P < 0.01
Temperature ^b^	16.7 (1.4)**	18.2 (1.2)**	28.7 (2.3)**	ns	P < 0.01
Wind ^b^	6.4 (2.2)**	4.1 (2.2)	-0.3 (3.4)	ns	ns
R^2^ within ^c^	1.5%	1.1%	1.4%		
Rho ^d^	11.3%	8.9%	8.3%		
F	3.5**	3.6**	3.3**		

^a^ CRF categories: CRF measured as VO_2peak_ (mL·kg^-1^·min^-1^) and categorized into unfit (25% lowest), moderately fit (50% medium) and highly fit (25% highest) individuals.

^b^ Precipitation; measured as mm, Temperature; measured as °C, Wind; measured as m·s^−1^.

^c^ R^2^ within; How much precipitation, temperature and wind explains of the variance in Overall PA

^d^ Rho; How much of the variance is due to difference across time (interclass correlation).

**Statistical significant (P < 0.01)

*Statistical significant (P < 0.05), ns Non-significant.

CRF: Cardiorespiratory fitness. PA: Physical activity measured as triaxial counts·min^-1^ (mean per hour).

### Weather and physical activity within colder months

For every additional °C, PA increased with 6-, 9- and 16 CPM among unfit, moderately fit and highly fit females, respectively (all, p<0.01) (Table 6). The influence of temperature was significantly greater among highly fit compared to unfit females (p<0.01). Precipitation did not influence PA in females during colder months, despite level of CRF.

**Table 6:** Regression coefficients of weather and physical activity in colder months, across gender and categories of cardiorespiratory fitness.

Total n = 631, 57055 hours	Weather and PA in “Colder” months (November–March)									
	in females, across CRF categories ^a^			CRF difference among females		in males, across CRF categories ^a^			CRF difference among males	
	Unfit (n = 80, 6034 hours)	Moderately fit (n = 148, 13527 hours)	Highly fit (n = 78, 7568 hours)	Unfit vs. moderately fit	Unfit vs. highly fit	Unfit (n = 81, 6442 hours)	Moderately fit (n = 165, 15823)	Highly fit (n = 79, 7661 hours)	Unfit vs. moderately fit	Unfit vs. highly fit
PA	509.3 (11.5)	614.6 (8.8)	691.5 (13.8)			453.6 (12.9)	578.9 (11.7)	693.4 (14.4)		
Precipitation ^b^	-10.8 (29.9)	-7.0 (22.3)	14.1 (36.9)	ns	ns	-17.7 (32.2)	59.0 (24.2)*	131.4 (38.7)**	P < 0.05	P < 0.01
Temperature ^b^	6.1 (1.7)^b^	9.3 (1.6)**	15.6 (2.5**	ns	P < 0.01	8.9 (2.4)**	8.6 (1.8)**	18.8 (3.3)**	ns	P < 0.01
Wind ^b^	-10.3 (4.6)*	-8.6 (3.3)**	-5.8 (5.4)	ns	ns	-3.9 (4.5)	-9.3 (4.7)*	-11.8 (5.6)*	ns	ns
R^2^ within ^c^	0.4%	0.4%	0.7%			0.4%	0.3%	0.7%		
Rho ^d^	12.4%	8.9%	6.2%			6.5%	6.2%	6.8%		
F	3.7**	3.2**	3.8**			3.7**	3.2**	3.8**		

^a^ CRF categories: CRF measured as VO2peak (mL·kg-1·min-1) and categorized into unfit (25% lowest), moderately fit (50% medium) and highly fit (25% highest) individuals.

^b^ Precipitation; measured as mm, Temperature; measured as °C, Wind; measured as m·s^−1^.

^c^ R^2^ within; How much precipitation, temperature and wind explains of the variance in Overall PA

^d^ Rho; How much of the variance is due to difference across time (interclass correlation).

**Statistical significant (P < 0.01)

*Statistical significant (P < 0.05), ns Non-significant.

CRF: Cardiorespiratory fitness. PA: Physical activity measured as triaxial counts·min^-1^ (mean per hour).

In males, PA increased with 59- and 131 CPM for every additional mm precipitation among moderately- (p<0.05) and highly (p<0.01) fit individuals, respectively. Precipitation did not influence PA among unfit males. For every additional °C, PA increased with 9-, 9-, and 19 CPM among unfit, moderately fit and highly fit males, respectively (all, p<0.01).

### Weather and physical activity within warmer months

In unfit females, PA decreased with 48 CPM for every additional mm precipitation (p<0.01) (Table 7). Precipitation did not influence moderately- and highly fit females. For every additional °C, PA increased with 27-, 28- and 50 CPM among unfit, moderately fit and highly fit females, respectively (all, p<0.01).

**Table 7:** Regression coefficients of weather and physical activity in warmer months, across gender and categories of cardiorespiratory fitness.

Total n = 588, 53833 hours	Weather and PA in “Warmer” months (April–October)									
	in females, across CRF categories ^a^			CRF difference among females		in males, across CRF categories ^a^			CRF difference among males	
	Unfit (n = 94, 8111 hours)	Moderately fit (n = 151, 14605 hours)	Highly fit (n = 73, 6541 hours)	Unfit vs. moderately fit	Unfit vs. highly fit	Unfit (n = 91, 7251 hours)	Moderately fit (n = 115, 11188 hours)	Highly fit (n = 64, 6137 hours)	Unfit vs. moderately fit	Unfit vs. highly fit
PA	282.6 (24)	365.3 (27.3)	351.8 (41.2)			245.6 (26.9)	333.2 (26.9)	346.9 (39.6)		
Precipitation ^b^	-47.6 (16.9)**	-30.3 (20.0)	-13.4 (24.9)	ns	ns	-39.8 (22.4)*	-25.6 (23.2)	49.8 (66.4)	ns	ns
Temperature ^b^	26.5 (3.0)**	28.1 (4.2)**	49.6 (5.3)**	ns	P < 0.01	27.7 (3.5)**	35.2 (3.6)**	46.7 (6.4)**	ns	P < 0.01
Wind ^b^	14.5 (3.6)**	19.2 (3.9)**	13.2 (8.3)	ns	ns	11.6 (4.2)**	14.6 (5.7)*	9.7 (9.2)	ns	ns
R^2^ within ^c^	3.5%	3.0%	3.6%			3.0%	2.7%	4.4%		
Rho ^d^	13.9%	9.4%	8.2%			6.7%	9.4%	8.4%		
F	3.9**	3.2**	3.7**			3.9**	3.9**	3.1**		

^a^ CRF categories: CRF measured as VO2peak (mL·kg-1·min-1) and categorized into unfit (25% lowest), moderately fit (50% medium) and highly fit (25% highest) individuals.

^b^ Precipitation; measured as mm, Temperature; measured as °C, Wind; measured as m·s^−1^.

^c^ R^2^ within; How much precipitation, temperature and wind explains of the variance in Overall PA

^d^ Rho; How much of the variance is due to difference across time (interclass correlation).

**Statistical significant (P < 0.01)

*Statistical significant (P < 0.05), ns Non-significant.

CRF: Cardiorespiratory fitness. PA: Physical activity measured as triaxial counts·min^-1^ (mean per hour).

In unfit males, PA decreased for every additional mm of precipitation (p<0.05). Moderately- and higly fit males were not influenced by precipitation. For every additional °C, PA increased with 28-, 35-, and 48 CPM for unfit, moderately fit and highly fit males, respectively (all, p<0.01). The influence was significantly greater in highly fit males compared to unfit males (p<0.01).

## Discussion

To our knowledge, this is the first study examining how the hour-to-hour and seasonal weather relate to PA among older adults. Independently of season, gender and level of CRF, precipitation did not influence PA among older adults, while temperature had a positive influence. Weather explained substantially more of the variance in PA in warmer months compared to colder months. Increasing temperatures in both warmer and colder months had a positive influence on PA, and this influence was even higher for fit participants compared to those unfit. Increasing precipitation in warmer months had a negative influence on PA for unfit females and unfit males. Increasing precipitation in colder months, however, had a positive influence on PA in moderately and highly fit males, but not in unfit males and females.

In accordance with previous studies, the present study found older adults to be less physically active in colder months (November to March) compared to warmer months (April to October) [8, 21]. Season is, however, a “crude” measure when it comes to understanding the effect weather on PA. Weather varies considerably from day-to-day and from hour-to-hour, particularly in temperate climates. In addition, by examining hour-to-hour changes as a complement to seasonal differences, possible seasonal confounders are eliminated. [18].

Weather in the present study explained about the same percentage of variance in PA (0.4–4.4) compared to other studies. In a study of Australian adults using pedometers, they found the variance to range between 0–5.8% depending on type of PA, where weather variations showed the best explanatory power for moderate and vigorous PA [17]. In a study on Dutch older adults using a GPS logger, weather (+covariates) was found to explain 0.043% and 0.028% of walking and cycling activity, respectively [18]. These findings clearly highlight the importance of acknowledging different PA barriers when developing PA interventions for older adults.

Our findings are quite similar to those from European countries (UK, Netherland and Germany), especially the positive association between increasing temperatures and PA [18–21]. As illustrated in a qualitative study examining barriers and facilitators of PA among older US adults, weather might actually be a facilitator for PA (“*I’m kind of a sunshine walker”*) [14]. Adding to this body of knowledge, our study shows that the positive influence temperature has on PA (hour-to-hour) is stronger for fit participants than for unfit participants.

Furthermore, while European studies (18–21) have reported that increasing precipitation was negatively associated with PA, our study found this association to be dependent on the level of CRF. I.e. while fit older adults (females and males) were physically active regardless of precipitation in warmer months, this seemed to be a barrier for the unfit individuals. Additionally, we found precipitation to positively influence PA in fit and moderately fit males during colder months. This is in line with the findings in Chan et al. [16], who reported that leaner Canadian males became more physically active when it snowed compared to males with high body mass index (BMI), who reduced their PA [16]. Based on our results, we argue that fit older adults have a greater commitment to PA compared to unfit participants, and hence would be more likely to be active in unpleasant weather [16, 22]. These findings highlight the importance of acknowledging individual`s level of CRF when examining how weather influence PA-level.

The authors acknowledge that it isn`t possible to differentiate time spent (performing PA) outdoors and indoors in the current study. However, qualitative studies reported that fear of falling and concerns over safety are potential barriers for outdoor PA among older adults [14, 38, 39]. Poor weather conditions might augment these concerns and thereby reduce outdoor PA among this age group. Notably, outdoor recreational walking is the most common PA type among older adults in Norway [13]. This might be a reasonable explanation as to why the weather, in the current study, explained more of the variance in PA in warmer versus the colder months. In colder months in Norway, older adults might perform more of their daily PA indoors, due to generally poorer weather conditions, higher probability of icy surfaces, and less daylight.

While it is not possible to change the weather conditions, a better understanding of how weather influences PA might be helpful when developing strategies to ameliorate the impact of adverse weather conditions on future PA interventions in older adults. Importantly, the current study clearly shows that PA in less fit older adults is more likely to suffer as a result of poor weather conditions compared to PA of their fit counterparts. Results from the current study suggest that unfit individuals should be an important target group when developing strategies that could reduce the negative impact of poor weather conditions on PA in older adults (e.g. indoor leisure facilities, better transport links (accessibility) and safe surfaces for walking etc.) [12, 40].

### Strengths and limitations

One of the strengths of this study was the large sample of older adults with varying fitness (10.1–52.8 mL·kg^-1^·min^-1^) and PA levels. Moreover, the use of objectively- and simultaneously measured PA- and weather data (190 000 hours), gave us the opportunity to compare hourly change in weather and older adults’ PA level. The use of panel data and fixed effects regression models to analyze the impact of time varying weather data on older adults’ PA is a major attempt to establish a causal relationship. Such a design, where the same units of analysis were recorded over time, makes causal analyses more trustworthy compared to investigating cross-sectional data.

A limitation to the present study is that it did not assess data related to the surface (i.e. wet or slippery), which might influence PA in older adults [14]. Furthermore, since PA was obtained with accelerometers, the present study cannot determine whether the activities were performed indoors or outdoors, which is highly relevant when examining the influence of weather. Future studies should therefore aim to implement type and context of activities (log/diary) when examining the effect of weather on PA.

It is important to acknowledge that weather, in the current study, only explained 2% (Colder months: 0.5%, warmer months: 5%) of the variance in PA (R^2^ within). Hence, most of the variance in PA is unexplained. However, finding the interclass correlation (Rho) to explain 9% of the variance due to differences across time, highlights the value of having a panel data design which emphasizes a longitudinal dimension.

Our findings were from a group of relatively healthy older adults (70–77 years) [24], living in the municipality of Trondheim, Norway, with its local weather characteristics and may not be generalizable to other populations. However, in general, this study contributes to a better understanding of how weather might influence PA in older adults across season, gender and fitness.

## Conclusions

Our findings suggest that fit older adults would likely be more willing to accept some unpleasant weather when being active, while their unfit counterparts might experience bad weather as a barrier towards PA. Importantly, the impact of weather shouldn`t be ignored when planning public health strategies for increasing PA among older adults, especially for those who are unfit.
